# An Anti-Parkinson’s Disease Drug via Targeting Adenosine A_2A_ Receptor Enhances Amyloid-β Generation and γ-Secretase Activity

**DOI:** 10.1371/journal.pone.0166415

**Published:** 2016-11-11

**Authors:** Jing Lu, Jin Cui, Xiaohang Li, Xin Wang, Yue Zhou, Wenjuan Yang, Ming Chen, Jian Zhao, Gang Pei

**Affiliations:** 1 State Key Laboratory of Cell Biology, Institute of Biochemistry and Cell Biology, Shanghai Institutes for Biological Sciences, Chinese Academy of Sciences, Shanghai, 200031, China; 2 Graduate School, University of Chinese Academy of Sciences, 320 Yueyang Road, Shanghai, 200031, China; 3 School of Life Science and Technology, ShanghaiTech University, 100 Haike Road, Shanghai, 201210, China; 4 Chemical Biology Core Facility, Institute of Biochemistry and Cell Biology, Shanghai Institutes for Biological Sciences, Chinese Academy of Sciences, Shanghai, 200031, China; 5 Translational Medical Center for Stem Cell Therapy, Shanghai East Hospital, School of Medicine, Tongji University, Shanghai, 200120, China; 6 School of Life Science and Technology, Collaborative Innovation Center for Brain Science, Tongji University, Shanghai, 200092, China; Sungkyunkwan University, REPUBLIC OF KOREA

## Abstract

γ-secretase mediates the intramembranous proteolysis of amyloid precursor protein (APP) and determines the generation of Aβ which is associated with Alzheimer’s disease (AD). Here we identified that an anti-Parkinson’s disease drug, Istradefylline, could enhance Aβ generation in various cell lines and primary neuronal cells of APP/PS1 mouse. Moreover, the increased generation of Aβ_42_ was detected in the cortex of APP/PS1 mouse after chronic treatment with Istradefylline. Istradefylline promoted the activity of γ-secretase which could lead to increased Aβ production. These effects of Istradefylline were reduced by the knockdown of A_2A_R but independent of A_2A_R-mediated G protein- or β-arrestin-dependent signal pathway. We further observed that A_2A_R colocalized with γ-secretase in endosomes and physically interacted with the catalytic subunit presenilin-1 (PS1). Interestingly, Istradefylline attenuated the interaction in time- and dosage-dependent manners. Moreover the knockdown of A_2A_R which in theory would release PS1 potentiated both Aβ generation and γ-secretase activity. Thus, our study implies that the association of A_2A_R could modulate γ-secretase activity. Istradefylline enhance Aβ generation and γ-secretase activity possibly via modulating the interaction between A_2A_R and γ-secretase, which may bring some undesired effects in the central nervous system (CNS).

## Introduction

AD is a most common neurodegenerative disorder causing progressive memory loss and cognitive impairment. Mounting evidence indicates that one of the major pathological hallmarks of AD is the accumulation of Aβ plaques composed of two major Aβ peptides, Aβ_40_ and Aβ_42_ [1]. Aβ is produced by the sequential cleavage of APP by β-secretase and γ-secretase complex consisting of PS1, nicastrin (NCT), anterior pharynxdefective phenotype 1 (APH1) and presenilin enhancer 2 (Pen2) [2–5]. PS1 is the catalytic subunit of the complex and its mutations account for a large amount of familial AD (FAD) cases [6]. Several endogenous modulators of γ-secretase have been reported that include transmembrane trafficking protein 21-KD [7], the γ-secretase-activating protein [8], CD147 antigen [9], and G protein-coupled receptors (GPCRs). Notably, GPCRs could modulate secretase activities via signal transductions or their interactions with secretase components [10–13]. GPCRs are abundantly expressed in CNS and function as the major therapeutic targets for many neurological disorders [14, 15]. Whether these GPCRs or their targeting medications could modulate γ-secretase activity or Aβ generation requires further investigation.

A_2A_R, belonging to Family A GPCRs, are widely expressed in the CNS including striatum, hippocampus, and cortex and play essential roles in the regulation of locomotion, sleep, anxiety, memory, and cognition [16, 17]. Recently, A_2A_R has emerged as a non-dopaminergic target for the treatment of PD, owing to its physical and functional interaction with dopamine D_2_ receptor in striato-pallidal GABA pathway [18]. Istradefylline, a selective A_2A_R antagonist and an approved anti-PD drug in Japan, efficiently crosses blood-brain barrier, binds to A_2A_R with high affinity, and potentiates L-DOPA (a dopamine precursor; standard of PD therapeutics) activity [19]. Notably, dementia is detected in some cases of PD with abnormal accumulation of Aβ [20–22]. Whether the anti-PD drugs could modulate Aβ generation is worth investigation. In the present study, we identified Istradefylline as a modulator of Aβ generation through targeting A_2A_R. A_2A_R interacts with PS1 of γ-secretase complex and modulates γ-secretase activity. Binding with Istradefylline to the receptor may attenuate the interaction, leading to a more ‘condensed’ conformation of PS1 and an increased secretase activity for Aβ generation.

## Materials and Methods

### Animals

The animal experiments were performed according to the National Institutes of Health Guide for the Care and Use of Laboratory Animals. The related protocols were approved by the Biological Research Ethics Committee, Shanghai Institutes for biological Sciences, Chinese Academy of Sciences. Animal pain and discomfort were minimized with efforts. APP/PS1 double-transgenic mice (The Jackson Laboratory, USA, stock number 004462) expressing a chimeric mouse/human APPswe and a human PS1 with exon-9 deletion (PS1ΔE9) were maintained and genotyped according to the guidance of Jackson Laboratory. These mice display an aggressive onset of age-dependent neuritic Aβ deposition in the cortex and hippocampus from six months of age. Six month-old, age- and gender-matched APP/PS1 mice were evenly grouped to vehicle- or Istradefylline-treated groups (2 mouse/cage) and subjected to the oral gavage of vehicle solution or Istradefylline (3 mg/kg/day, dissolved in saline with 5% Tween-80) daily. None mouse became severely ill during the experiment. Brain samples were collected for Aβ_42_ and Aβ_40_ analyses after drug administration.

### Materials

Ligands ZM 241385 and SCH 442416 were purchased from Sigma (St Louis, MO, USA). Preladenant and Tozadenant were obtained from MedChem Express (Monmouth Juncton, NJ, USA). Other receptor ligands were from Selleck Chemicals (Houston, TX, USA). Fluorogenic substrate for γ-secretase was from Calbiochem (Hayward, CA, USA). All other chemicals and reagents used were purchased from Sigma (St Louis, MO, USA) unless otherwise indicated.

### Antibodies

Anti-PS1-N-terminus (1–65) (PRB-354P, 1:1,000, Covance, Davis, CA, USA); Anti-PS1-N-terminus (MAB1563, 1:100, EMD Millipore, Darmstadt, Germany); anti-PS1 loop (263–407) (529592, 1:2,000, Calbiochem); anti-APH1aL/C terminal (245–265) (PRB-550P, 1:1,000, Covance); anti-NCT (N1660, 1:1,000, Sigma), Anti-Pen2 (P5622, 1:1,000, Sigma); anti-BACE1 (AP7774b, 1:1,000, Abgent, Suzhou, China); anti-HA (H6908, 1:5,000, Sigma); anti-Flag (F3156, 1:2,000, Sigma); anti-A_2A_R (05–717, 1:1,1000, Millipore); anti-EEA1 (610457, dilution 1:200, BD transduction laboratories).

### Plasmids and siRNA

The cDNA sequences of human APH1aL, NCT, PS1 and Pen2 were subjected to codon optimization and cloned into pcDNA3 vector to generate pAPH1aL, pNCT, pPS1 and pPen2 plasmids with varying tags (Life Technologies, USA). The pMLink vector was kindly provided by Prof. Yigong Shi (Tsinghua University, Beijing, China). Human Pen2, NCT and APH1aL were individually cloned into the pMLink vector at the multiple cloning sites to generate pMLink-Pen2-NCT-APH1aL. A_2A_R plasmid was a generous gift from Prof. Xin Xie (Shanghai Institute of Materia Medica, Shanghai, China). It was then cloned into 5’Flag pcDNA3 vector or used as template for site-directed mutagenesis following an overlapping PCR approach. The mutants were also cloned into 5’Flag pcDNA3 vectors. For fluorescent-labeled constructs, CFP or YFP was fused to the N-terminus or C-terminus of each protein with a 12 amino acid linker, GSGGGGSGGGGS, in between and cloned into p3639 vector. Transfection was performed using Effectene Transfection Reagent (QIAGEN, Hilden, Germany) for all cells for 48–72 h.

Two siRNA duplex were used to knockdown A_2A_R. The sequences were: 5'-CCUAAGGGAAG GAGAUCUUUA(dT)(dT)-3' and 5'-UGCUCAUG CUGGGUGUCUAUU (dT)(dT)-3'. The siRNA sequences of β-arrestin1 and 2 were: 5'-GGAAGCUCAAGCACGAAGACTT-3' and 5’-GGCTTGTCCTTCCGCAAAGACA-3’. The oligos were synthesized and purified by GenePharma (Shanghai, China). SiRNA or plasmid/siRNA co-ransfection was performed using Effectene Transfection Reagent for HEK293, HEK293T or HEK293/APPswe cells or X-tremeGENE siRNA transfection reagent (Roche, Basel, Switzerland) for SH-SY5Y cells.

### Cell culture

All cell lines used here were originally purchased from ATCC. HEK293/APPswe cells were transfected, selected with antibiotics (G418) and maintained in lab. HEK293, HEK293T and HEK293/APPswe were cultured in MEM, and SH-SY5Y in MEM/F12 with 10% (*v/v*) heat-inactivated fetal bovine serum (FBS) in a humidified incubator with 5% CO2/95% air (*v/v*) at 37°C.

### Primary culture

The preparation of mouse primary neuronal cells was performed according to the standard protocols [23, 24] with minor modification. Briefly, after dissection of the cortices and hippocampi from APP/PS1 P0 pups, cells were trypsinized, dissociated, and then seeded into 96-well plates. The neuronal cells were maintained in 1*B27 and 1*Glutamax (Gibco, 35050)-containing Neurobasal medium. The medium was half-refreshed every 4 days and chemical treatment was performed on DIV8.

### Reverse Transcription and Quantitative Real-Time PCR

Total RNA was extracted with TRI Reagent (T9424; Sigma) according to the manufacturer’s instructions. Random hexamer primer and MMLV Reverse Transcriptase (M5301; Promega) were used for reverse transcription. All gene transcripts were quantified by quantitative real-time PCR performed with 2 × HotStart SYBR Green qPCR Master Mix (ExCell Bio, Shanghai, China) on a Stratagene Mx3000P (Agilent Technologies). The primer used for the detection of mRNA level of A_2A_R, β-arrestin1 and β-arrestin2 were as below: human A_2A_R sense: 5’-CATGCTAGGTTGGAACAACTGC-3’; anti-sense: 5’-AGATCCGCAAATAGACACCCA-3’; human β-arrestin1 sense: 5’-GGGACCCGAGTGTTCAAGAA-3’; anti-sense: 5’-ACAAACAGGTCCTTGCGAAAG-3’; human β-arrestin2 sense: 5’-GTCGAGCCCTAACTGCAAG-3’; anti-sense: 5’-ACAAACACTTTGCGGTCCTTC-3’. All the primers were synthesized and purified by Shanghai Sunny Biotechnology Co., Ltd.

### ELISA for Aβ

HEK293/APPswe or SH-SY5Y cells were cultured in 96- or 48-well plates and treated with chemicals at indicated concentrates for 2 h or 24 h for the detection of Aβ levels. The media was then collected and subject to sandwich ELISA assay for the detection of total Aβ following the manufacturer’s instruction. The ELISA kit was from ExCell Bio (Shanghai, China).

### Secretase activity assays based on the fluorogenic substrate

This experiment is performed according to the previous publications [25, 26]. HEK293T cells with indicated transfection and/or 2 h chemical treatment were lysed in buffer A (25 mM Tris-HCl, 5 mM EDTA, 5 mM EGTA, adjusted to pH 7.4) and centrifuged to remove debris and nuclei. The membrane fractions were enriched by ultracentrifuge and resuspended in reaction buffers (including 10 μM of specific fluorogenic substrate, without or with the presence of indicated chemicals). After incubation at 37°C for 120 min, fluorescence of the cleaved substrates was measured by SpectraMax M5 spectrometer (Molecular Devices).

### cAMP assay

The intracellular cAMP was measured using GloSensor^TM^ cAMP assay following the manufacturer’s instruction with minor modification (Promega, Madison, WI, USA). HEK293/APPswe or CHO/APPswe cells were seeded in 96-well plates (Costar Cat. #3917) and transfected with pGloSensor^TM^-22F cAMP plasmid using Effectene Transfection reagent. Before the cAMP assay, the media was removed and replaced with the fresh medium containing 2% (*v/v*) of GloSensor^TM^ cAMP reagent. After 90 min incubation at 37°C with 5% CO_2_, cells were equilibrated at room temperature (RT) for 20 min, and treated with the ligands at indicated concentrations for 15 min, followed by the measurement of luciferase activity.

### Immuonfluoresence microscopy

HEK293 cells grown on cover-slip were transfected with required plasmids for 48 h and then treated with indicated chemicals followed by fixation with 4% paraformaldehyde (PFA) in PBS for 10 min. Cells were permeabilized and blocked with PBS/0.2% Triton X-100/1% BSA for 45 min. Cells were then incubated with indicated primary antibodies for 2 h at RT. After washing with PBS/1% BSA for three times, cells were incubated with Cy3-labeled goat anti-mouse or rabbit IgG secondary antibodies in the dark for 1 h, washed with PBS/1% BSA, and mounted on slides. Images were acquired using LAS SP8 confocal microscope (Leica, Germany) with a 63 ×/1.40 NA oil objective (Leica).

For the analysis of receptor internalization, the acquired images were subject to the measurement of fluorescence intensity at regions of plasma membrane and cytosol using ImageJ (http://rsb.info.nih.gov/ij/). The index of receptor internalization was then calculated according to the published equation [27]

To quantify the degree of colocalization between fluorophores, the images were background subtracted and subjected to the analysis of Mander’s colocalization coefficients using ImageJ (http://rsb.info.nih.gov/ij/).

### Acceptor Photobleaching *fluorescence resonance energy transfer* (FRET) assay

The assay was performed following the reported methods [28, 29]. HEK293 cells were seeded in 24-well plate with cover-slips and transfected. After 48 h, cells were treated as indicated and fixed with 4% PFA for 10 min, washed with PBS for three times, and mounted on slides. Samples were subjected to acceptor photobleaching FRET imaging with a confocal microscope (LAS SP8; Leica) with a 63×/1.40 NA oil objective (Leica). Image acquisition, registration, background subtraction and data analyses were performed with Leica Application Suite Advanced Fluorescence (LAS AF) software. Imaging conditions were set up manually: CFP (excitation: 405 nm, emission: 465–505 nm) and YFP (excitation: 514 nm, emission: 525–600 nm). Photobleach was performed using 514-nm light and over 70% bleach efficiency was achieved. Images of CFP and YFP channels were acquired pre- and post-bleaching. FRET efficiency was calculated as percentage of enhancement in donor fluorescence (f) after acceptor photobleaching:

E = 1-f[CFP(pre)] / f[CEP(post)]. Five non-bleached regions were selected and the average values were used to correct the FRET efficiency of photobleached region.

### FRET SE

Cells were transfected with CFP alone (donor only) or YFP alone (acceptor only) or co-transfected with CFP-PS1 and A_2A_R-YFP (sample). 48 h later, cells were treated with 30 nM of Istradefylline for indicated times. Cells were then fixed and subjected to sensitized emission FRET assay. The images of donor only and acceptor only in three channels, donor, FRET and acceptor, were taken prior to the test of samples. The ROI net intensity was used to generate the relative correction parameters as shown in the formula below. For the samples, the images were simultaneously obtained in channels of CFP, FRET and YFP as the selection of ROI. The fluorescence density of each channel was background subtracted. The FRET efficiency was calculated with the formula: E = (B–A × β –C × (γ – α × β)) / (C × (1 – β × δ)). A, B, C correspond to the intensities of the 3 channels (donor, FRET, acceptor). α, β, γ and δ are the calibration factors generated by acceptor only and donor only references [30].

### Co-immunoprecipitation (co-IP)

The assay was carried out as previously reported [11, 29]. In brief, 48 h post transfection HEK293T cells were treated with chemicals as indicated for 30 min. Total cell lysates were lysed with IP buffer (50 mM HEPES pH 7.4, 150 mM NaCl, 10% Glycerol, 1% CHAPSO or 1% TritonX-100). Cell lysates were incubated with anti-Flag M2 resins at 4°C for 4 h. The resins were washed three times and eluted with SDS loading buffer before Western blotting analysis. Whole brains of APP/PS1 mice were homogenized by a glass dounce tissue grinder in Buffer A (25 mM Tris-HCl, 5 mM EDTA, 5 mM EGTA, adjusted to pH 7.4) and centrifuged to remove debris and nuclei. After centrifuged at 25,000 × g for 1 h, 600 mg membrane proteins were resuspended in IP buffer and incubated with antibodies at 4°C for 16 h. For each mouse sample, equal amount of membrane fractions were incubated with 2 μg Goat anti-rat IgG or MAB1563. The antibody-antigen complexes were then incubated with pre-equilibrated Ezview Red Protein G Affinity Gel beads for 1 h at 4°C. After washed, the resins were eluted with SDS loading buffer for Western blotting analysis.

### Statistic analysis

All experiments were repeated at least three times. Data are representative or mean +/± SEM. All data were analyzed by Prism 6.0 (GraphPad Software Inc., San Diego, CA). Unpaired Student’s t-test (two-tailed) was applied for the comparisons of two groups. One-way or Two-way analysis of variance (ANOVA) with Bonferroni’s post-test for multiple comparisons, or Dunnett’s post-test to compare each group with a single control group was used where more than two groups were compared. Statistical significance was accepted at *p* < 0.05.

## Results

### Istradefylline was identified to promote Aβ generation

**Fig 1:**
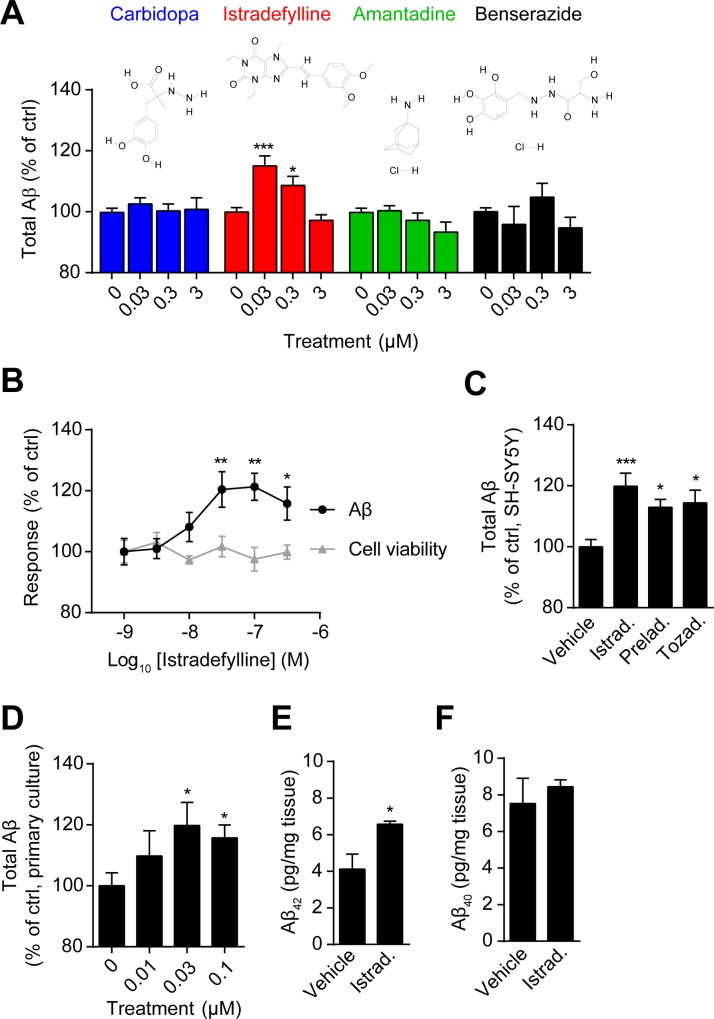
Istradefylline promotes Aβ generation. (A) HEK293/APPswe cells were treated with Carbidopa, Istradefylline, Amantadine or Beserazide at indicated concentrations for 2 h followed by the detection of total Aβ levels in the culture media. The chemical structure of each compound is presented. (B) The secreted total Aβ levels and cell viability in response to Istradefylline at a range of concentrations. (C) The endogenous Aβ production of SH-SY5Y cells upon the treatment with 0.1% of DMSO (Vehicle), Istradefylline (Istrad.), Preladenant (Prelad.) or Tozadenant (Tozad.) at 30 nM for 24 h was examined. (D) The primary neuronal culture of APP/PS1 mouse was stimulated with vehicle (0) or Istradefylline at indicated concentrations for 24 h followed by the measurement of Aβ. T-test was used here. (E) The level of Aβ_42_ in the cortex of APP/PS1 transgenic mouse after chronic treatment with vehicle or Istradefylline. *N* = 11/group. (F) The level of Aβ_40_ in the cortex of APP/PS1 transgenic mouse after chronic treatment with vehicle or Istradefylline. *N* = 11/group. Data are mean +/± SEM of at least three independent experiments. *, *p* < 0.05; **, *p* < 0.01; ***, *p* < 0.001.

We firstly examined the cellular Aβ generation in response to the treatments with anti-PD drugs in HEK293/APPswe cells. Carbidopa and Benserazide inhibit the activity of dopamine decarboxylase and prevent the degradation of levodopa, the precursor of dopamine, in the peripheral tissues. They are often used clinically for the treatment of PD in the combination with levodopa. Amantadine is a weak antagonist of the NMDA-type glutamate receptor, increasing dopamine release and blocking its re-uptake [31]. Istradefylline, a selective antagonist of A_2A_R, is approved for clinical use in Japan and currently under global phase 3 trial [19]. We found that Carbidopa, Amantadine or Beserazide showed little effect on the cellular production of total Aβ (Fig 1A). However, Istradefylline significantly enhanced Aβ production in a dosage-dependent manner with no obvious change of cell viability (Fig 1B). Moreover, Istradefylline increased the endogenous Aβ generation in a human neuroblastoma cell line, SH-SY5Y (Fig 1C). We also examined the effects of other A_2A_R-targeting anti-PD agents on Aβ modulation. Preladenant has failed in the clinical test and Tozadenant is now under clinical trial [32]. Similar with Istradefylline, both of them increased the endogenous Aβ production in SH-SY5Y cells (Fig 1C).

We further examined the effect of Istradefylline in primary neuronal culture of APP/PS1 mouse. Istradefylline consistently enhanced the generation of Aβ in a dosage-dependent manner (Fig 1D). Furthermore, we studied whether Istradefylline could modulate Aβ generation *in vivo*. An increase of Aβ_42_ in the cortices of APP/PS1 mice was detected after the chronic treatment with Istradefylline (Fig 1E) while there is no significant change of Aβ_40_ (Fig 1F). Meanwhile, in the mouse hippocampi, no significant change of either Aβ_42_ or Aβ_40_ was observed (data not shown).

### Istradefylline promotes Aβ generation and γ-secretase activity through A_2A_R

**Fig 2:**
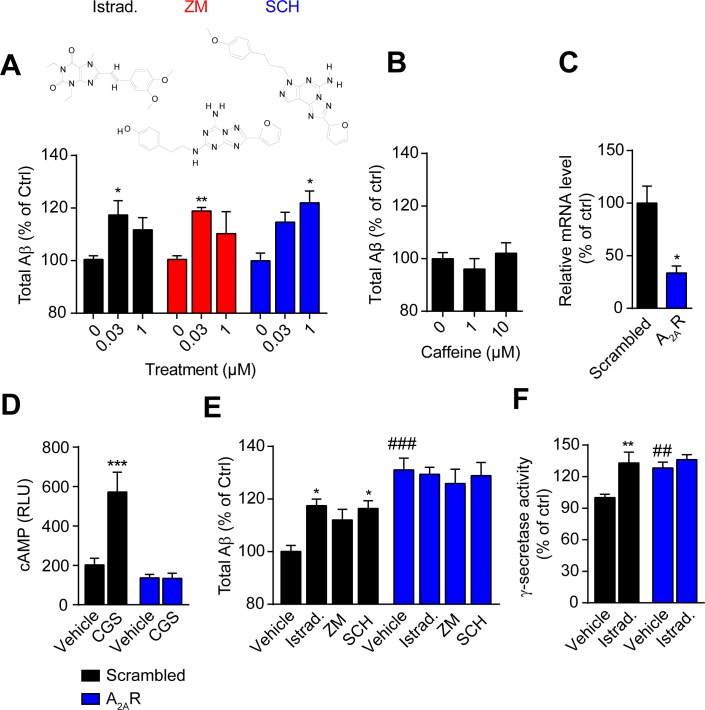
Istradefylline promotes Aβ generation and γ-secretase activity through targeting A_2A_Rs. (A) A_2A_R antagonists increase the total Aβ level. HEK293/APPswe cells were treated with Istradefylline (Istrad.), ZM 241385 (ZM) or SCH 442416 (SCH) at indicated concentrations for 2 h and the total Aβ productions were then determined. The chemical structure of each compound is shown. (B) Caffeine-modulated total Aβ generation in HEK293/APPswe cells. Cells were treated with caffeine at 1 or 10 μM for 2 h followed by the determination of total Aβ levels in the culture media. (C) Quantification of A_2A_R mRNA level after transfection with scrambled or A_2A_R siRNA in HEK293/APPswe. (D) The cAMP response of the cells to agonist treatment. HEK293/APPswe cells were co-transfected with pGloSensor^TM^-22F cAMP plasmid and scrambled or A_2A_R siRNA followed by the stimulation with 1% of DMSO (Ctrl) or 30 nM of CGS 21680 HCl (CGS). Data are shown in arbitrary luminescence units. (E) The knockdown of A_2A_R reduces antagonists-increased Aβ generation. Cells transfected with scrambled or A_2A_R siRNA were treated with 0.1% of DMSO (Vehicle), Istradefylline (Istrad.), ZM 241385 (ZM) or SCH 442416 (SCH) at 30 nM followed by the determination of total Aβ. (F) The knockdown of A_2A_R reduces Istradefylline-increased γ-secretase activity. Cells transfected with scrambled or A_2A_R siRNA were stimulated with vehicle or Istradefylline (Istrad.) at 30 nM followed by the measurement of γ-secretase activity. Data are mean + SEM of at least three independent experiments. *, *p* < 0.05; **, *p* < 0.01; ***, *p* < 0.001 vs. control within the group. ##, *p* < 0.01; ###, *p* < 0.001 vs. control of scambled.

We then performed a range of assays to explore the underlying mechanisms. Istradefylline is a selective A_2A_R antagonist. Thus, we reasoned that Istradefylline might modulate Aβ promotion via targeting A_2A_R. First, we examined the Aβ production in response to other two highly selective A_2A_R antagonists, ZM 241385, a selective A_2A_R antagonist sharing similar chemical core structure with Istradefylline, and SCH 442416, a non-xanthine derivative antagonist. Similar to Istradefylline, ZM 241385 and SCH 442416 also increased Aβ generation in HEK293/APPswe cells (Fig 2A). By contrast, the non-selective A_2A_R antagonist caffeine did not influence Aβ production (Fig 2B). Then we investigated whether the Istradefylline-modulated Aβ increase could be prevented by the knockdown of A_2A_R. Transfection with siRNA targeting A_2A_R in SH-SY5Y cells expressing APPswe (SH-SY5Y/APPswe) successfully reduced the mRNA level of A_2A_R quantified by real-time PCR (Fig 2C). Functional experiment revealed that interfering with A_2A_R expression hugely reduced the cellular cAMP level in response to the agonist CGS 21680 HCl stimulation (Fig 2D), indicating the efficiency of knockdown. Data showed that knockdown of A_2A_R itself promoted Aβ generation and in this context, Istradefylline, ZM 241385 or SCH 442416-modulated Aβ increase was completely blocked (Fig 2E). Ligand-bound A_2A_R crystal structures reveal that amino acids Phe168 in the second extracellular loop and Asn253 in the sixth intramembrane helix are critical for ligand binding [33–35]. The individual expression of two Ala substitution mutants of A_2A_R, F168A and N253A, prevented CGS 21680 HCl-stimulated cAMP response without the influence of receptor expression or distribution (S1A and S1B Fig). CHO cells are reported to have relatively low expression of A_2A_R and thus are commonly used to introduce recombinant receptors for their functional studies [36]. Here we did not detect any obvious change of Aβ generation in A_2A_R ligands-treated CHO/APPswe cells (S1C Fig, β-gal). However, in the cells expressing wild-type A_2A_Rs, antagonists Istradefylline, ZM 241385 or SCH 442416 significantly increased Aβ level (S1C Fig, WT). All these effects were prevented by the expression of F168A or N253A mutant (S1C Fig, F168A or N253A).

Receptors could regulate Aβ generation via the modulation of secretase activity [11–13]. Here we found that Istradefylline significantly enhanced γ-secretase activity (Fig 2F) while it had little effect on α- or β-secretase activity (data not shown). Meanwhile, knockdown of A_2A_R promoted the activity of γ-secretase (Fig 2F), but not α- or β-secretase (data not shown). The increased γ-secretase activity upon A_2A_R knockdown was not further promoted by Istradefylline treatment indicating Istradefylline increases γ-secretase activity via A_2A_R (Fig 2F). These data elucidated that antagonizing or silencing A_2A_R could increase γ-secretase activity and Aβ production.

### Istradefylline-modulated Aβ promotion is independent of G protein- or β-arrestin1/2-dependent signal pathway

**Fig 3:**
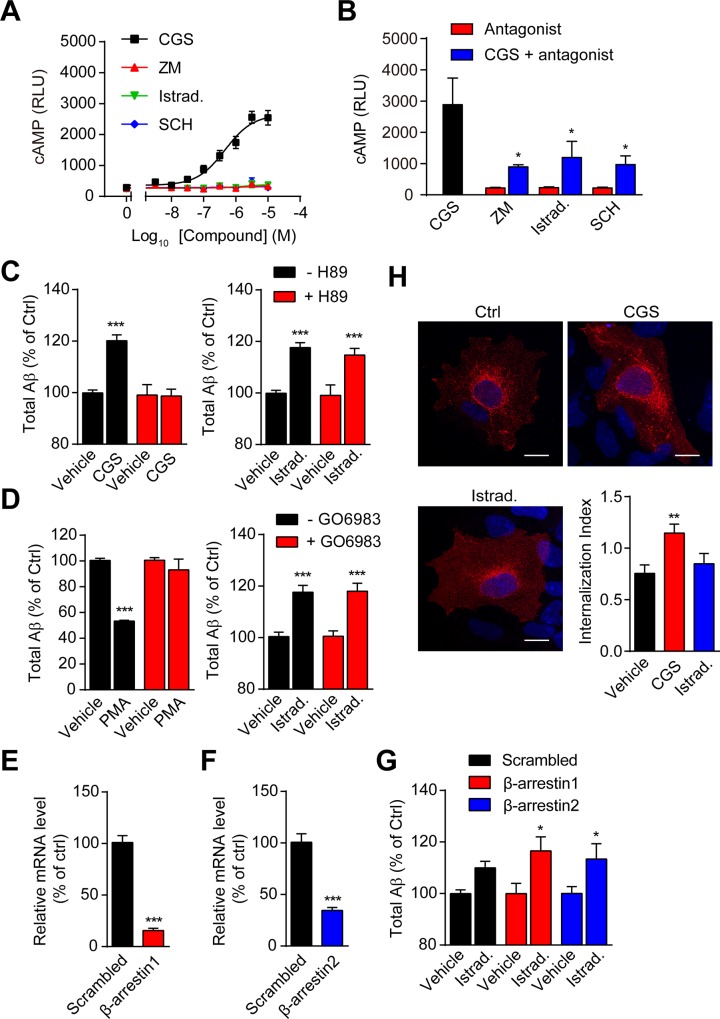
The increased Aβ generation induced by Istradefylline is not through PKA, PKC or β-arrestins. (A) A_2A_R ligands-modulated cAMP response in HEK293/APPswe cells. Cells were transfected with pGloSensor^TM^-22F cAMP plasmid and then stimulated with CGS 21680 HCl (CGS), ZM 241385 (ZM), Istradefylline (Istrad.) or SCH 442416 (SCH) at indicated concentrations. (B) A_2A_R antagonists block agonist-induced cAMP increase. Cells were pre-treated with ZM 241385 (ZM, 1 nM), Istradefylline (Istrad., 100 nM) or SCH 442416 (SCH, 1 nM) followed by the stimulation with CGS 21680 HCl (CGS, 1 μM). (C) The effect of A_2A_R ligands on Aβ production in the absence or presence of H89. Cells were incubated without or with 10 μM of H89, prior to the treatment with 0.1% of DMSO (Ctrl), CGS 21680 HCl (CGS, left) or Istradefylline (Istrad., right). H89 was present in the indicated groups throughout the experiments. (D) The effect of PKC activator or Istradefylline on Aβ production in the absence or presence of GO6983. HEK293/APPswe cells were pre-treated without or with 1 μM of PKC inhibitor GO6983 followed by the treatment with 0.1% of DMSO (Ctrl), 1 μM of PMA (left) or 30 nM of Istradefylline (Istrad., right). (E-G) The effect of β-arrestin1 or 2 on Istradefylline-modulated increase of Aβ generation. (E, F) Quantification of mRNA levels after transfection with scrambled, β-arrestin1 (E) or β-arrestin2 (F) siRNA in HEK293/APPswe cells. (G) After the transfection with scrambled, β-arrestin1 or β-arrestin2 siRNA, HEK293/APPswe cells were subjected to the treatment with 0.1% of DMSO (Ctrl) or Istradefylline (30 nM) followed by the detection of secreted total Aβ levels. (H) Representative images and the accompanied analysis of A_2A_R endocytosis upon the treatment with 0.1% of DMSO (Ctrl), CGS 21680 HCl (CGS) or Istradefylline (Istrad.) at 1 μM in HEK293 cells transfected with Flag-A_2A_R. Scale bar = 10 μm. *N* = 18–24 cells. Data are representative or mean +/± SEM of at least three independent experiments. *, *p* < 0.05; **, *p* < 0.01; ***, *p* < 0.001. PMA, phorbol 12-myristate 13-acetate.

GPCRs could modulate secretase activity through cAMP/PKA- or PKC-regulated signal pathway [13]. A_2A_R conducts biological functions mainly via the activation of cAMP/PKA signal pathway with evidence indicating the involvement of PKC pathway in the modulation of macrophage functions [37]. Although Istradefylline is an antagonist of A_2A_R, it is also a xanthine derivative. Xanthine and its analogs have the potential to inhibit the activity of phosphodiesterase and increase the level of intracellular cAMP [38]. Here, we verified that Istradefylline did not trigger a cAMP response but predominantly inhibited agonist CGS 21680 HCl-induced cAMP increase (Fig 3A and 3B). We further introduced PKA or PKC inhibitor to determine the role of signal transduction in Istradefylline-modulated Aβ generation. We found that the pre-incubation with PKA inhibitor H89 in HEK293/APPswe cells fully inhibited the effect upon agonist CGS 21680 HCl (Fig 3C) stimulation demonstrating the inhibitor was efficient. However, it did not influence Istradefylline-modulated Aβ promotion. Furthermore, PKC inhibitor GO6983 significantly prevented PMA (PKC activator)-modulated Aβ reduction in HEK293/APPswe cells but had no discernible effect on Istradefylline-regulated Aβ generation (Fig 3D). β-arrestins mediate receptor endocytosis which have been reported to involve in the modulation of Aβ production [39, 40]. The associations of β-arrestin1 and 2 with A_2A_R have been demonstrated [41]. We asked if Istradefylline could modulate Aβ generation through β-arrestins. Knockdown of β-arrestin1 or 2 predominantly reduced its mRNA level (Fig 3E and 3F), but did not block Istradefylline-increased Aβ production (Fig 3G). Additionally, immuno-staining of Flag-A_2A_R revealed that Istradefylline was unable to promote receptor endocytosis which was significantly enhanced by the treatment with the agonist CGS 21680 HCl (Fig 3H). The above data suggested that Istradefylline-modulated increase of Aβ generation was independent of G protein- or β-arrestin1/2-mediated signal pathway.

### A_2A_R interacts with γ-secretase complex

**Fig 4:**
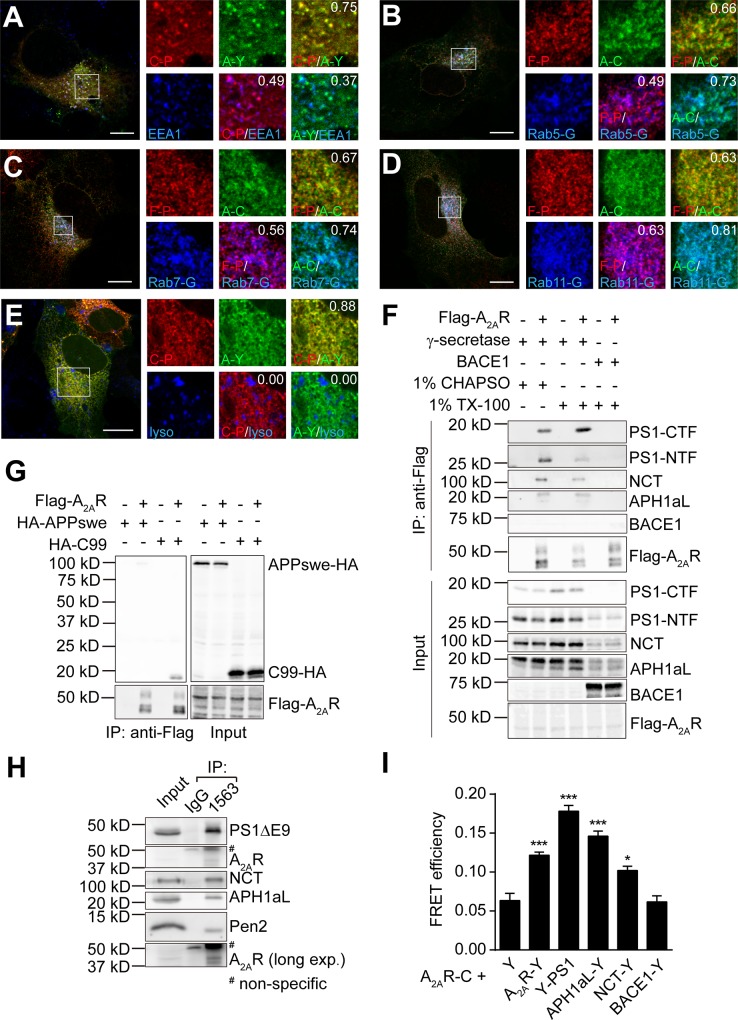
A_2A_R colocalizes with γ-secretase complex in endosomes and interacts with PS1. (A-E) Representative images showing the subcellular distribution of PS1 and A_2A_R with endocytic markers. HEK293 cells were co-transfected with indicated protein constructs and pMLink-Pen2-NCT-APH1aL and stained for EEA1 or Flag-PS1 or labeled with lysostracker. (A) Subcellular colocalization of CFP-PS1 (C-P), A_2A_R-YFP (A-Y) and EEA1. (B-D) Subcellular colocalization of Flag-PS1 (F-P), A_2A_R-CFP (A-C) and Rab5-GFP (Rab5-G, B), Rab7-GFP (Rab7-G, C), Rab11-GFP (Rab11-G, D). (E) Subcellular colocalization of CFP-PS1 (C-P), A_2A_R-YFP (A-Y) and lysotracker (lyso). Scale bar = 10 μm. White box indicates the enlarged area. *N* ≥ 10 cells for each group. The number on the right-top corner presents the Mander’s colocalization coefficiency of two channels. (F) Representative image showing co-IP of γ-secretase complex components or BACE1 with Flag-A_2A_R in HEK293T cells. Cells, transfected with four γ-secretase complex components (PS1, NCT, APH1aL and Pen2) or BACE1 and β-gal or Flag-A_2A_R, were lysed in the buffer containing 1% of CHAPSO or 1% of TritonX-100 (TX-100) and immunoprecipitated with anti-Flag slurry. The antibodies were previously verified [11]. (G) Flag-A_2A_R co-immunoprecipitates with HA-C99, but not HA-APPswe. Cells were transfected with HA-APPswe or HA-C99 without or with Flag-A_2A_R and then lysed, immunoprecipitated and blotted. Representative image is shown. (H) A_2A_R interacts with PS1 in APP/PS1 mouse brain. Brain membrane fractions were extracted from a 9 month APP/PS1 mouse. PS1 was immunoprecipitated with anti-PS1 antibody (1563 as labeled in the figure) and samples were subjected to Western-blot analysis. The antibody recognizing the endogenous A_2A_R has been verified [58]. (I) FRET efficiency of A_2A_R-CFP with YFP-fused other proteins. HEK293 cells were transfected with A_2A_R-CFP and YFP alone (Y), A_2A_R-YFP (A_2A_R-Y), APH1aL-YFP (APH1aL-Y), NCT-YFP (NCT-Y), YFP-PS1 (Y-PS1) or BACE1-YFP (BACE1-Y). Cells were then fixed and subjected to acceptor photobleaching FRET experiment. *N* ≥ 30 cells per condition. Data are mean + SEM. *, *p* < 0.05; ***, *p* < 0.001.

Some GPCRs have been found to modulate γ-secretase activity via their interactions [11, 12]. We hypothesized A_2A_R could also interact with γ-secretase complex. To begin with, we explored the subcellular distribution of A_2A_R and γ-secretase complex by immunofluorescence imaging. HEK293 cells were transfected with A_2A_R-YFP and four γ-secretase components (CFP-PS1, NCT, APH1aL, and Pen2). Mild expression of A_2A_R-YFP or CFP-PS1 was observed at plasma membrane (S2 Fig). Dynasore, a dynamin inhibitor, was then used to accumulate A_2A_R-YFP or γ-secretase at plasma membrane. As suspected a relatively higher expression of plasma membrane A_2A_R-YFP was detected while the colocalization was week. In cytosolic compartments, A_2A_R-YFP displayed punctate patterns and colocalized with CFP-PS1. The cells were further stained with subcellular compartment markers. Alternatively, the complex components (Flag-PS1, NCT, APH1aL, and Pen2), A_2A_R-CFP, and GFP-tagged Rab5, 7 or 11 were transfected to monitor their colocalization. CFP- or Flag-tagged PS1 colocalized with early endosome markers (EEA1 and Rab5-GFP, Fig 4A and 4B), a late endosome marker (Rab7-GFP, Fig 4C), and a recycling endosome marker (Rab11-GFP, Fig 4D), consistent with the distribution of endogenous γ-secretase complex [42]. Meanwhile, A_2A_R-YFP or A_2A_R-CFP largely distributed in the cytosolic compartments colocalizing with CFP-PS1 or Flag-PS1 respectively in a punctate pattern and presented in EEA1-, Rab5-, Rab7- or Rab11-labeled compartments. By contrast, we did not observe clear distribution of colocalized A_2A_R and PS1 in LysoTracker-labeled lysosomes (Fig 4E). The accompanied analyses of Mander’s colocalization coefficients demonstrated consistent results. These data showed that A_2A_Rs colocalize with γ-secretase complexes in endosomes.

Next, we verified the interaction of A_2A_R with γ-secretase components using co-IP assay. 1% CHAPSO- or 1% TritonX-100-soluble cell lysates were prepared from HEK293T cells over-expressing four γ-secretase components (PS1, NCT, APH1aL and Pen2) or BACE1, without or with Flag-tagged A_2A_R. PS1 undergoes endoproteolytic process to generate an N- terminal and C-terminal fragment (PS1-NTF and PS1-CTF respectively) which form a complex [43]. In 1% CHAPSO-soluble cell lysates, γ-secretase components PS1-CTF, PS1-NTF, NCT and APH1aL were all co-immunoprecipitated with Flag-A_2A_R, indicating A_2A_R interacts with intact γ-secretase complex (Fig 4F, Lane 1 and 2). In 1% Triton X-100-containing buffer which dissociates the γ-secretase complex, less amount of PS1-NTF, NCT and APH1aL were detected, while the amount of co-immunoprecipitated PS1-CTF was even enriched (Fig 4F, Lane 3 and 4). Meanwhile, we did not detect any obvious association of BACE1 with Flag-A_2A_R (Fig 4F, Lane 5 and 6). APP is cleaved by β-secretase and produces C99 which is the direct substrate of γ-secretase. GPCR was reported to interact with APP and modulate its distribution and processing [10]. Here we did not detect the co-IP of APPswe-HA with Flag-A_2A_R in 1% TritonX-100 soluble lysates while a weak interaction between C99-HA and A_2A_R was observed (Fig 4G). Notably, the interaction between A_2A_R and PS1 was also detected in the brain of 9 months old APP/PS1 mouse, suggesting the interaction was detectable under pathological conditions (Fig 4H).

The interaction between cytosolic A_2A_R and PS1 was further quantified using FRET technique. A_2A_R has been reported to dimerize [44]. Here we used A_2A_R-CFP and A_2A_R-YFP as a positive control for acceptor photobleaching FRET assay. Compared to the negative control (A_2A_R-CFP and YFP alone, ~ 0.05 of FRET efficiency), A_2A_R-CFP and A_2A_R-YFP conducted a significant FRET efficiency (~ 0.12) (Fig 4I). In this context, we detected a robust FRET efficiency between A_2A_R-CFP and YFP-PS1 (~ 0.17). The FRET efficiency between A_2A_R-CFP and APH-1aL-YFP was slightly lower but higher than that between A_2A_R-CFP and NCT-YFP. A_2A_R-CFP and BACE1-YFP-conducted FRET efficiency was ~ 0.05, which was close to the negative control (Fig 4I). Collectively, the above data suggested that A_2A_R had close proximity with γ-secretase complex.

### Istradefylline attenuates the interaction between A_2A_R and PS1 and influences the internal conformation of PS1

**Fig 5:**
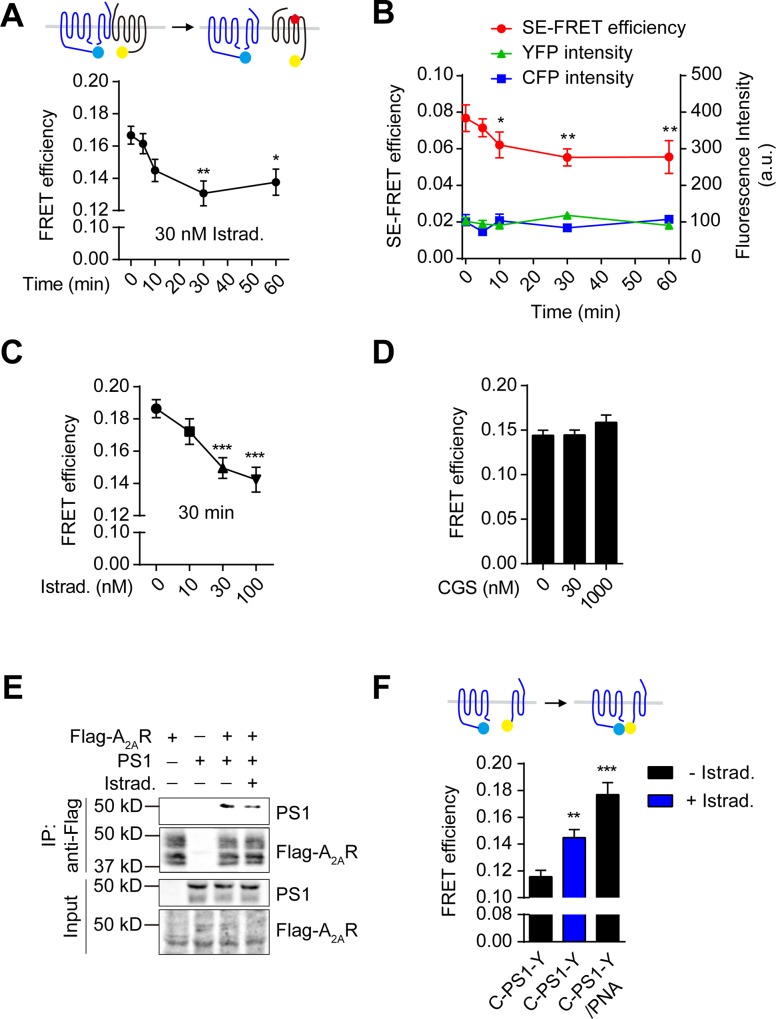
Istradefylline, but not CGS 21680 HCl, attenuates the interaction between A_2A_R and PS1 and influences the internal conformation of PS1. (A, B) HEK293 cells co-transfected with CFP-PS1 and A_2A_R-YFP were treated with Istradefylline (30 nM) for 0–60 min followed by the determination of FRET efficiency using acceptor photobleaching FRET (A) or FRET SE technique (B). Both the FRET efficiency and the attribute units of YFP or CFP intensity are presented in B. *N* ≥ 20 cells per condition. a.u., arbitrary units. Alternatively, the transfected cells were treated with Istradefylline (C) or CGS21680 HCl (D) at indicated concentrations for 30 min followed by acceptor photobleaching FRET assay. (E) Representative image showing Istradefylline reduces the co-IP of PS1 with Flag-A_2A_R. HEK293T cells were transfected with Flag-A_2A_R alone, PS1 alone or co-transfected with Flag-A_2A_R and PS1. Cells were treated without or with 30 nM of Istradefylline and then lysed with 1% of TritonX-100 IP buffer, followed by immunoprecipitation and western blotting. (F) PS1 conformation revealed by the internal FRET efficiency of CFP-PS1-YFP. HEK293 cells were transfected with CFP-PS1-YFP (C-PS1-Y) followed by the treatment without or with 30 nM of Istradefylline. As a control, cells were co-transfected with CFP-PS1-YFP (C-PS1-Y) and pMLink-Pen2-NCT-APH1aL (PNA). *N* ≥ 30 cells per condition for FRET assays. Data are mean +/± SEM. *, *p* < 0.05; **, *p* < 0.01; ***, *p* < 0.001.

Istradefylline is a neutral antagonist binding to the receptor without influencing its basal activity which could lead to receptor constitutive endocytosis [45–47]. The ligand could possibly enter into endosome compartments via receptor constitutive endocytosis to modulate the physical association of receptor with γ-secretase, thereby influencing its activity for Aβ generation. We performed FRET and co-IP assay to investigate the effects of ligands on the interaction between A_2A_R and PS1. A_2A_R was reported to modulate cell migration [48] and we did spot rapid and profound cell movements upon ligand treatment. Thus, we applied acceptor-bleached or sensitized-emission FRET (FRET SE) assay in the fixed cells. Both experiments consistently showed that Istradefylline treatment reduced FRET efficiency between CFP-PS1 and A_2A_R-YFP in a time-dependent manner with a maximum effect after 30 min (Fig 5A and 5B). The fluorescence intensities of CFP and YFP in the selected areas for measurement were consistent at each time points. Furthermore, Istradefylline also showed dosage-dependence on the modulation of the interaction (Fig 5C). Treatment with 10 nM of Istradefylline had little effect on the FRET efficiency between A_2A_R-CFP and YFP-PS1 while 30 nM or 100 nM treatment significantly reduced the signal. Notably, the agonist CGS 21680 HCl had little effect on the FRET efficiency between A_2A_R-CFP and YFP-PS1 (Fig 5D). We also observed that Istradefylline attenuated the interaction of PS1 with Flag-A_2A_R in co-IP (Fig 5E). The above results indicated that Istradefylline attenuates the interaction between A_2A_R and PS1 in time- and dosage-dependent manners.

Internal FRET probe of PS1 reveals its conformational changes under different physiological or pathological conditions and correlates with the γ-secretase activity [49, 50]. We asked if the Istradefylline-modulated interaction of A_2A_R with PS1 may lead to a conformational change of PS1, thereby influencing its activity. As reported [28], CFP-PS1-YFP conducted an efficient FRET efficiency which was potentiated by the co-expression of APH1aL, NCT and Pen2, accompanied with increased γ-secretase activity. Here we consistently detected the increased FRET efficiency of CFP-PS1-YFP in the presence of APH1aL, NCT and Pen2 (Fig 5F). Moreover, upon the treatment with Istradefylline, the FRET efficiency of CFP-PS1-YFP was also significantly enhanced, indicating an altered PS1 conformation which may contribute to the increased γ-secretase activity (Fig 5F).

## Discussion

A_2A_R is highly expressed in striatum and modulates dopaminergic neurotransmission to control motor activity. Thus it has been a therapeutic target for the treatment of PD in combination with dopaminergic medications. A_2A_R also presents at a lower level in cortex and hippocampus where regulate cognitive functions. However the role of A_2A_R on cognition is controversial [51]. For example, there is evidence showing the genetic inactivation of A_2A_R reverses working memory deficits at early stages of Huntington’s disease while another study demonstrating the genetic blockage of A_2A_R induces cognitive impairments in schizophrenia animal model [52, 53]. It is possible that A_2A_R differentially regulates cognitive functions under different disease conditions. In this study, we identified that Istradefylline, an A_2A_R antagonist used for PD therapy, could increase Aβ generation in various cells including primary neuronal cells of AD mouse model. Moreover, the increased level of Aβ_42_ in cortex of APP/PS1 mouse implying a potential undesired effect of A_2A_R antagonists in AD. However, the non-selective adenosine receptor antagonist caffeine had little effect which would not compromise its protective effect in an AD mouse model [54]. Interestingly, we did not observed the effect of Istradefylline on Aβ generation in hippocampus indicating the effect is region or cell-type specific. This could be due to the distinct expression, distribution or downstream machinery of A_2A_R. Moreover, A_2A_R could interact with other GPCRs such as D_2_R or possibly γ-secretase as presented in the current study. The effect of Istradefylline could also depend on the expression or distribution of these interacting proteins in a specific region or cell. These may further indicate a complicated mechanism of A_2A_R on cognition.

**Fig 6:**
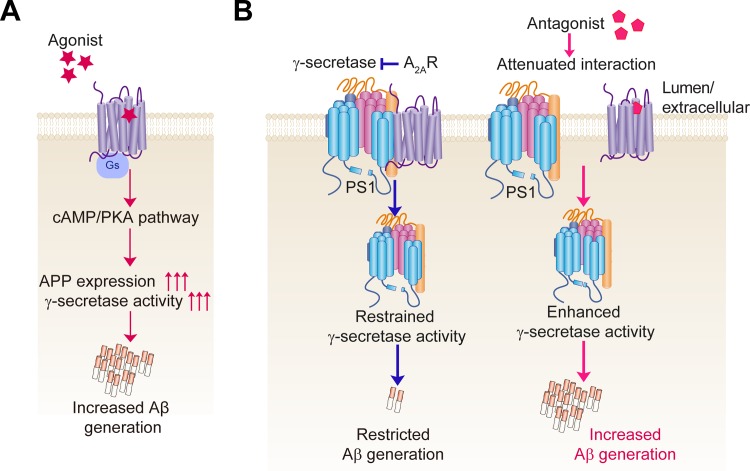
Scheme of A_2A_R modulates γ-secretase activity and Aβ generation. (A) Reported scheme of A_2A_R agonist promoting Aβ production. Agonist binding to the receptor activates Gs protein leading to the activation of cAMP/PKA signal pathway. The elevated cAMP promotes APP expression and γ-secretase activity resulting into increased Aβ generation. (B) A scheme of A_2A_R antagonist-modulating Aβ generation revealed by our study. A_2A_R forms a complex with γ-secretase complex composed of PS1 (blue), APH1 (red), NCT (orange) and Pen2 (dark blue), and binds with PS1-CTF, restraining γ-secretase activity for Aβ production. Antagonist binding-induced conformational change of A_2A_R may attenuate the association with γ-secretase complex resulting in a “condensed” state of PS1, favoring Aβ generation.

Accumulating evidences show that besides G protein-mediated signal pathway GPCRs could enhance Aβ generation via their interactions with the secretases that modulate APP processing [11, 12, 40], leading to the re-distribution of the complex and increased secretase activity for substrate proteolysis. Interestingly, though both agonist and antagonist of A_2A_R enhance Aβ production in a receptor-dependent manner, their underlying mechanisms are distinct (Fig 6). The former depends on G_s_ protein-mediated signal pathway while the latter is independent of A_2A_R-mediated G protein- or β-arrestin-dependent signal pathway. The reduced interaction between A_2A_R and PS1 by Istradefylline treatment indicates that Istradefylline may regulate Aβ generation and γ-secretase activity via modulating the interaction between A_2A_R and γ-secretase complex. The knockdown of A_2A_R leads to increased Aβ generation and enhanced γ-secretase activity suggesting the association of A_2A_R could regulate γ-secretase activity for Aβ generation. A proper modulation of this interaction by ligand binding may achieve the reduction of Aβ generation which requires further investigation. Thus, in addition to modulating the interaction between A_2A_R and D_2_R for PD treatment, our study suggests that A_2A_R ligands could also modulate GPCR-secretase interaction to regulate secretase activity for substrate processing, which may provide a novel target for AD.

Clinical analyses reported the association between memory disorders and the chronic administration of drugs including antidepressants, anticonvulsants, and notably an anti-PD drug, Artane [55]. Artane is an M1 muscarinic acetylcholine receptor (M1 mAChR) antagonist and M1 mAChR is reported to involve in Aβ pathology *in vivo* [56]. Interestingly, we observed that Artane also increased cellular Aβ production (data not shown), while the underlying mechanism remains to be clarified. Among those antidepressants and anticonvulsants, many of them are known to target the corresponding GPCRs and it might be worthy to investigate if they affect Aβ pathology. Dementia is well-recognized in patients with PD. Thus, the anti-PD drugs improving both motor and memory deficits would be ideal. Although Istradefylline has been reported to improve cognitive performance in a 6-hyroxydopamine-lesioned PD model [57], an elevated generation of Aβ in an AD model as observed in the present study would bring some concerns. Considering the genetic difference between mouse and human, a clinical analysis assessing the correlation between memory disorder and chronic treatment with Istradefylline might be beneficial.

## Supporting Information

S1 Fig Istradefylline promotes Aβ generation in CHO/APPswe cells expressing A_2A_Rs. (A) The cAMP levels in CHO/APPswe cells over-expressing wild type or mutant A_2A_Rs. Cells were co-transfected with pGloSensor^TM^-22F cAMP plasmid and β-galactosidase (β-gal), wild type-A_2A_R (WT), mutant F168A or N253A followed by the treatment with 0.1% of DMSO (Ctrl) or 30 nM of CGS 21680 HCl. (B) Representative image showing the cellular distribution of wild type-A_2A_R and its mutants in HEK293 cells. Cells were transfected with Flag-tagged wild type-A_2A_R (WT), F158A or N253A followed by immuno-staining. Scale bar = 10 μm. (C) ligand-modulated Aβ production in the cells expressing wild type-A_2A_R or its binding mutants. CHO/APPswe cells were transfected with β-galactosidase (β-gal), wild type-A_2A_R (WT), mutant F168A or N253A followed by the treatment with 0.1% of DMSO (Ctrl) or the indicated ligands at 30 nM. Data are representative or mean + SEM of at least three independent experiments. **, *p* < 0.01; ***, *p* < 0.001.
(TIF)

S2 Fig A_2A_R-YFP colocalizes with CFP-PS1 in the cytosol. HEK293 cells were transfected with A_2A_R-YFP and γ-secretase components including CFP-PS1, NCT, APH1aL, and Pen2. Cells were then incubated without or with 80 μM dynasore for 30 min followed by fixation and imaging. The arrowheads and arrows indicate the expression at the plasma membrane and in the cytosol respectively. Scale bar = 10 μm.
(TIF)

## References

[1] Kang J, Lemaire HG, Unterbeck A, Salbaum JM, Masters CL, Grzeschik KH, et al The precursor of Alzheimer's disease amyloid A4 protein resembles a cell-surface receptor. Nature. 1987;325(6106):733–6. 10.1038/325733a0 . 2881207

[2] Haass C, Kaether C, Thinakaran G, Sisodia S. Trafficking and proteolytic processing of APP. Cold Spring Harbor perspectives in medicine. 2012;2(5):a006270 10.1101/cshperspect.a006270 22553493 PMC3331683

[3] Sherrington R, Rogaev EI, Liang Y, Rogaeva EA, Levesque G, Ikeda M, et al Cloning of a gene bearing missense mutations in early-onset familial Alzheimer's disease. Nature. 1995;375(6534):754–60. Epub 1995/06/29. 10.1038/375754a0 . 7596406

[4] Yu G, Nishimura M, Arawaka S, Levitan D, Zhang L, Tandon A, et al Nicastrin modulates presenilin-mediated notch/glp-1 signal transduction and betaAPP processing. Nature. 2000;407(6800):48–54. 10.1038/35024009 . 10993067

[5] Francis R, McGrath G, Zhang J, Ruddy DA, Sym M, Apfeld J, et al aph-1 and pen-2 are required for Notch pathway signaling, gamma-secretase cleavage of betaAPP, and presenilin protein accumulation. Dev Cell. 2002;3(1):85–97. . 12110170 10.1016/s1534-5807(02)00189-2

[6] Vetrivel KS, Zhang YW, Xu H, Thinakaran G. Pathological and physiological functions of presenilins. Mol Neurodegener. 2006;1:4 10.1186/1750-1326-1-4 16930451 PMC1513131

[7] Chen F, Hasegawa H, Schmitt-Ulms G, Kawarai T, Bohm C, Katayama T, et al TMP21 is a presenilin complex component that modulates gamma-secretase but not epsilon-secretase activity. Nature. 2006;440(7088):1208–12. 10.1038/nature04667 . 16641999

[8] He G, Luo W, Li P, Remmers C, Netzer WJ, Hendrick J, et al Gamma-secretase activating protein is a therapeutic target for Alzheimer's disease. Nature. 2010;467(7311):95–8. 10.1038/nature09325 20811458 PMC2936959

[9] Zhou S, Zhou H, Walian PJ, Jap BK. CD147 is a regulatory subunit of the gamma-secretase complex in Alzheimer's disease amyloid beta-peptide production. Proceedings of the National Academy of Sciences of the United States of America. 2005;102(21):7499–504. 10.1073/pnas.0502768102 15890777 PMC1103709

[10] Nelson CD, Sheng M. Gpr3 stimulates Aβ production via interactions with APP and β-arrestin2. PLoS One. 2013;8(9):e74680 10.1371/journal.pone.0074680 24069330 PMC3771882

[11] Ni Y, Zhao X, Bao G, Zou L, Teng L, Wang Z, et al Activation of beta2-adrenergic receptor stimulates gamma-secretase activity and accelerates amyloid plaque formation. Nature medicine. 2006;12(12):1390–6. 10.1038/nm1485 . 17115048

[12] Teng L, Zhao J, Wang F, Ma L, Pei G. A GPCR/secretase complex regulates beta- and gamma-secretase specificity for Abeta production and contributes to AD pathogenesis. Cell Res. 2010;20(2):138–53. 10.1038/cr.2010.3 . 20066010

[13] Thathiah A, De Strooper B. The role of G protein-coupled receptors in the pathology of Alzheimer's disease. Nat Rev Neurosci. 2011;12(2):73–87. 10.1038/nrn2977 . 21248787

[14] Komatsu H. Novel Therapeutic GPCRs for Psychiatric Disorders. Int J Mol Sci. 2015;16(6):14109–21. 10.3390/ijms160614109 26101869 PMC4490542

[15] Nickols HH, Conn PJ. Development of allosteric modulators of GPCRs for treatment of CNS disorders. Neurobiol Dis. 2014;61:55–71. 10.1016/j.nbd.2013.09.013 24076101 PMC3875303

[16] Jastrzębska J, Nowak E, Smaga I, Bystrowska B, Frankowska M, Bader M, et al Adenosine (A)(2A)receptor modulation of nicotine-induced locomotor sensitization. A pharmacological and transgenic approach. Neuropharmacology. 2014;81:318–26. 10.1016/j.neuropharm.2014.03.002 . 24632528

[17] Shen HY, Chen JF. Adenosine A(2A) receptors in psychopharmacology: modulators of behavior, mood and cognition. Curr Neuropharmacol. 2009;7(3):195–206. 10.2174/157015909789152191 20190961 PMC2769003

[18] Fuxe K, Marcellino D, Genedani S, Agnati L. Adenosine A(2A) receptors, dopamine D(2) receptors and their interactions in Parkinson's disease. Mov Disord. 2007;22(14):1990–2017. 10.1002/mds.21440 . 17618524

[19] Dungo R, Deeks ED. Istradefylline: first global approval. Drugs. 2013;73(8):875–82. 10.1007/s40265-013-0066-7 . 23700273

[20] Kotzbauer PT, Cairns NJ, Campbell MC, Willis AW, Racette BA, Tabbal SD, et al Pathologic accumulation of α-synuclein and Aβ in Parkinson disease patients with dementia. Arch Neurol. 2012;69(10):1326–31. 10.1001/archneurol.2012.1608 22825369 PMC3616136

[21] Compta Y, Parkkinen L, Kempster P, Selikhova M, Lashley T, Holton JL, et al The significance of α-synuclein, amyloid-β and tau pathologies in Parkinson's disease progression and related dementia. Neurodegener Dis. 2014;13(2–3):154–6. 10.1159/000354670 24028925 PMC4194631

[22] Irwin DJ, Lee VM, Trojanowski JQ. Parkinson's disease dementia: convergence of α-synuclein, tau and amyloid-β pathologies. Nat Rev Neurosci. 2013;14(9):626–36. 10.1038/nrn3549 23900411 PMC4017235

[23] Kaech S, Banker G. Culturing hippocampal neurons. Nat Protoc. 2006;1(5):2406–15. 10.1038/nprot.2006.356 . 17406484

[24] Beaudoin GM, Lee SH, Singh D, Yuan Y, Ng YG, Reichardt LF, et al Culturing pyramidal neurons from the early postnatal mouse hippocampus and cortex. Nat Protoc. 2012;7(9):1741–54. 10.1038/nprot.2012.099 . 22936216

[25] Farmery MR, Tjernberg LO, Pursglove SE, Bergman A, Winblad B, Naslund J. Partial purification and characterization of gamma-secretase from post-mortem human brain. The Journal of biological chemistry. 2003;278(27):24277–84. Epub 2003/04/17. 10.1074/jbc.M211992200 . 12697771

[26] Gruninger-Leitch F, Schlatter D, Kung E, Nelbock P, Dobeli H. Substrate and inhibitor profile of BACE (beta-secretase) and comparison with other mammalian aspartic proteases. The Journal of biological chemistry. 2002;277(7):4687–93. Epub 2001/12/14. 10.1074/jbc.M109266200 . 11741910

[27] Coopman K, Huang Y, Johnston N, Bradley SJ, Wilkinson GF, Willars GB. Comparative effects of the endogenous agonist glucagon-like peptide-1 (GLP-1)-(7–36) amide and the small-molecule ago-allosteric agent "compound 2" at the GLP-1 receptor. J Pharmacol Exp Ther. 2010;334(3):795–808. 10.1124/jpet.110.166009 20507928 PMC2939672

[28] Wang X, Cui J, Li W, Zeng X, Zhao J, Pei G. γ-Secretase Modulators and Inhibitors Induce Different Conformational Changes of Presenilin 1 Revealed by FLIM and FRET. J Alzheimers Dis. 2015;47(4):927–37. 10.3233/JAD-150313 . 26401772

[29] Cui J, Wang X, Li X, Wang X, Zhang C, Li W, et al Targeting the gamma/beta-secretase interaction reduces beta-amyloid generation and ameliorates Alzheimer's disease-related pathogenesis. Cell Discovery. 2015;1(15021). 10.1038/celldisc.2015.21 PMC4860824 27462420

[30] van Rheenen J, Langeslag M, Jalink K. Correcting confocal acquisition to optimize imaging of fluorescence resonance energy transfer by sensitized emission. Biophys J. 2004;86(4):2517–29. 10.1016/S0006-3495(04)74307-6 15041688 PMC1304099

[31] Mizoguchi K, Yokoo H, Yoshida M, Tanaka T, Tanaka M. Amantadine increases the extracellular dopamine levels in the striatum by re-uptake inhibition and by N-methyl-D-aspartate antagonism. Brain Res. 1994;662(1–2):255–8. . 7859080 10.1016/0006-8993(94)90821-4

[32] Pinna A. Adenosine A2A receptor antagonists in Parkinson's disease: progress in clinical trials from the newly approved istradefylline to drugs in early development and those already discontinued. CNS Drugs. 2014;28(5):455–74. 10.1007/s40263-014-0161-7 . 24687255

[33] Xu F, Wu H, Katritch V, Han GW, Jacobson KA, Gao ZG, et al Structure of an agonist-bound human A2A adenosine receptor. Science. 2011;332(6027):322–7. 10.1126/science.1202793 21393508 PMC3086811

[34] Lebon G, Warne T, Edwards PC, Bennett K, Langmead CJ, Leslie AG, et al Agonist-bound adenosine A2A receptor structures reveal common features of GPCR activation. Nature. 2011;474(7352):521–5. 10.1038/nature10136 21593763 PMC3146096

[35] Jaakola VP, Griffith MT, Hanson MA, Cherezov V, Chien EY, Lane JR, et al The 2.6 angstrom crystal structure of a human A2A adenosine receptor bound to an antagonist. Science. 2008;322(5905):1211–7. 10.1126/science.1164772 18832607 PMC2586971

[36] Klotz KN, Hessling J, Hegler J, Owman C, Kull B, Fredholm BB, et al Comparative pharmacology of human adenosine receptor subtypes—characterization of stably transfected receptors in CHO cells. Naunyn Schmiedebergs Arch Pharmacol. 1998;357(1):1–9. . 9459566 10.1007/pl00005131

[37] De Ponti C, Carini R, Alchera E, Nitti MP, Locati M, Albano E, et al Adenosine A2a receptor-mediated, normoxic induction of HIF-1 through PKC and PI-3K-dependent pathways in macrophages. J Leukoc Biol. 2007;82(2):392–402. 10.1189/jlb.0107060 . 17505024

[38] Ukena D, Schudt C, Sybrecht GW. Adenosine receptor-blocking xanthines as inhibitors of phosphodiesterase isozymes. Biochem Pharmacol. 1993;45(4):847–51. . 7680859 10.1016/0006-2952(93)90168-v

[39] Liu X, Zhao X, Zeng X, Bossers K, Swaab DF, Zhao J, et al β-arrestin1 regulates γ-secretase complex assembly and modulates amyloid-β pathology. Cell Res. 2013;23(3):351–65. 10.1038/cr.2012.167 23208420 PMC3587707

[40] Thathiah A, Horré K, Snellinx A, Vandewyer E, Huang Y, Ciesielska M, et al β-arrestin 2 regulates Aβ generation and γ-secretase activity in Alzheimer's disease. Nat Med. 2013;19(1):43–9. 10.1038/nm.3023 . 23202293

[41] Burgueño J, Blake DJ, Benson MA, Tinsley CL, Esapa CT, Canela EI, et al The adenosine A2A receptor interacts with the actin-binding protein alpha-actinin. J Biol Chem. 2003;278(39):37545–52. 10.1074/jbc.M302809200 . 12837758

[42] Vetrivel KS, Cheng H, Lin W, Sakurai T, Li T, Nukina N, et al Association of gamma-secretase with lipid rafts in post-Golgi and endosome membranes. The Journal of biological chemistry. 2004;279(43):44945–54. 10.1074/jbc.M407986200 15322084 PMC1201506

[43] Levitan D, Lee J, Song L, Manning R, Wong G, Parker E, et al PS1 N- and C-terminal fragments form a complex that functions in APP processing and Notch signaling. Proc Natl Acad Sci U S A. 2001;98(21):12186–90. 10.1073/pnas.211321898 11593035 PMC59789

[44] Canals M, Burgueño J, Marcellino D, Cabello N, Canela EI, Mallol J, et al Homodimerization of adenosine A2A receptors: qualitative and quantitative assessment by fluorescence and bioluminescence energy transfer. J Neurochem. 2004;88(3):726–34. . 14720222 10.1046/j.1471-4159.2003.02200.x

[45] Scarselli M, Donaldson JG. Constitutive internalization of G protein-coupled receptors and G proteins via clathrin-independent endocytosis. J Biol Chem. 2009;284(6):3577–85. 10.1074/jbc.M806819200 19033440 PMC2635037

[46] Costa T, Herz A. Antagonists with negative intrinsic activity at delta opioid receptors coupled to GTP-binding proteins. Proc Natl Acad Sci U S A. 1989;86(19):7321–5. 2552439 10.1073/pnas.86.19.7321 PMC298053

[47] Milligan G, Bond RA. Inverse agonism and the regulation of receptor number. Trends Pharmacol Sci. 1997;18(12):468–74. . 9458695 10.1016/s0165-6147(97)01139-5

[48] Richard CL, Tan EY, Blay J. Adenosine upregulates CXCR4 and enhances the proliferative and migratory responses of human carcinoma cells to CXCL12/SDF-1alpha. Int J Cancer. 2006;119(9):2044–53. 10.1002/ijc.22084 . 16823836

[49] Uemura K, Lill CM, Li X, Peters JA, Ivanov A, Fan Z, et al Allosteric modulation of PS1/gamma-secretase conformation correlates with amyloid beta(42/40) ratio. PloS one. 2009;4(11):e7893 10.1371/journal.pone.0007893 19924286 PMC2773935

[50] Dolev I, Fogel H, Milshtein H, Berdichevsky Y, Lipstein N, Brose N, et al Spike bursts increase amyloid-β 40/42 ratio by inducing a presenilin-1 conformational change. Nat Neurosci. 2013;16(5):587–95. 10.1038/nn.3376 . 23563578

[51] Wei CJ, Li W, Chen JF. Normal and abnormal functions of adenosine receptors in the central nervous system revealed by genetic knockout studies. Biochim Biophys Acta. 2011;1808(5):1358–79. 10.1016/j.bbamem.2010.12.018 . 21185258

[52] Li W, Silva HB, Real J, Wang YM, Rial D, Li P, et al Inactivation of adenosine A2A receptors reverses working memory deficits at early stages of Huntington's disease models. Neurobiol Dis. 2015;79:70–80. 10.1016/j.nbd.2015.03.030 . 25892655

[53] Moscoso-Castro M, Gracia-Rubio I, Ciruela F, Valverde O. Genetic blockade of adenosine A2A receptors induces cognitive impairments and anatomical changes related to psychotic symptoms in mice. Eur Neuropsychopharmacol. 2016;26(7):1227–40. 10.1016/j.euroneuro.2016.04.003 . 27133030

[54] Arendash GW, Schleif W, Rezai-Zadeh K, Jackson EK, Zacharia LC, Cracchiolo JR, et al Caffeine protects Alzheimer's mice against cognitive impairment and reduces brain beta-amyloid production. Neuroscience. 2006;142(4):941–52. 10.1016/j.neuroscience.2006.07.021 . 16938404

[55] Chavant F, Favrelière S, Lafay-Chebassier C, Plazanet C, Pérault-Pochat MC. Memory disorders associated with consumption of drugs: updating through a case/noncase study in the French PharmacoVigilance Database. Br J Clin Pharmacol. 2011;72(6):898–904. 10.1111/j.1365-2125.2011.04009.x 21557759 PMC3244635

[56] Davis AA, Fritz JJ, Wess J, Lah JJ, Levey AI. Deletion of M1 muscarinic acetylcholine receptors increases amyloid pathology in vitro and in vivo. The Journal of neuroscience: the official journal of the Society for Neuroscience. 2010;30(12):4190–6. 10.1523/JNEUROSCI.6393-09.2010 20335454 PMC2855655

[57] Kadowaki Horita T, Kobayashi M, Mori A, Jenner P, Kanda T. Effects of the adenosine A2A antagonist istradefylline on cognitive performance in rats with a 6-OHDA lesion in prefrontal cortex. Psychopharmacology (Berl). 2013;230(3):345–52. 10.1007/s00213-013-3158-x . 23748382

[58] Valadas JS, Batalha VL, Ferreira DG, Gomes R, Coelho JE, Sebastião AM, et al Neuroprotection afforded by adenosine A2A receptor blockade is modulated by corticotrophin-releasing factor (CRF) in glutamate injured cortical neurons. J Neurochem. 2012;123(6):1030–40. 10.1111/jnc.12050 . 23057965

