# Correction: An Anti-Parkinson's Disease Drug via Targeting Adenosine A_2A_ Receptor Enhances Amyloid-β Generation and γ-Secretase Activity

**DOI:** 10.1371/journal.pone.0172775

**Published:** 2017-02-16

**Authors:** The PLOS ONE Staff

Several of the grant numbers in the Funding section are listed incorrectly. The correct funding information is as follows: This research was supported by the Ministry of Science and Technology of China (2015CB964502, 2014CB964802), the National Natural Science Foundation of China (31371419), Science and Technology Commission of Shanghai Municipality (14DZ1900402). The publisher apologizes for the errors.
