# Supplementary figures and images for: An Anti-Parkinson’s Disease Drug via Targeting Adenosine A2A Receptor Enhances Amyloid-β Generation and γ-Secretase Activity
Source: PLoS One. 2016 Nov 11;11(11):e0166415. doi: 10.1371/journal.pone.0166415 (PMC5106031; doi:10.1371/journal.pone.0166415)

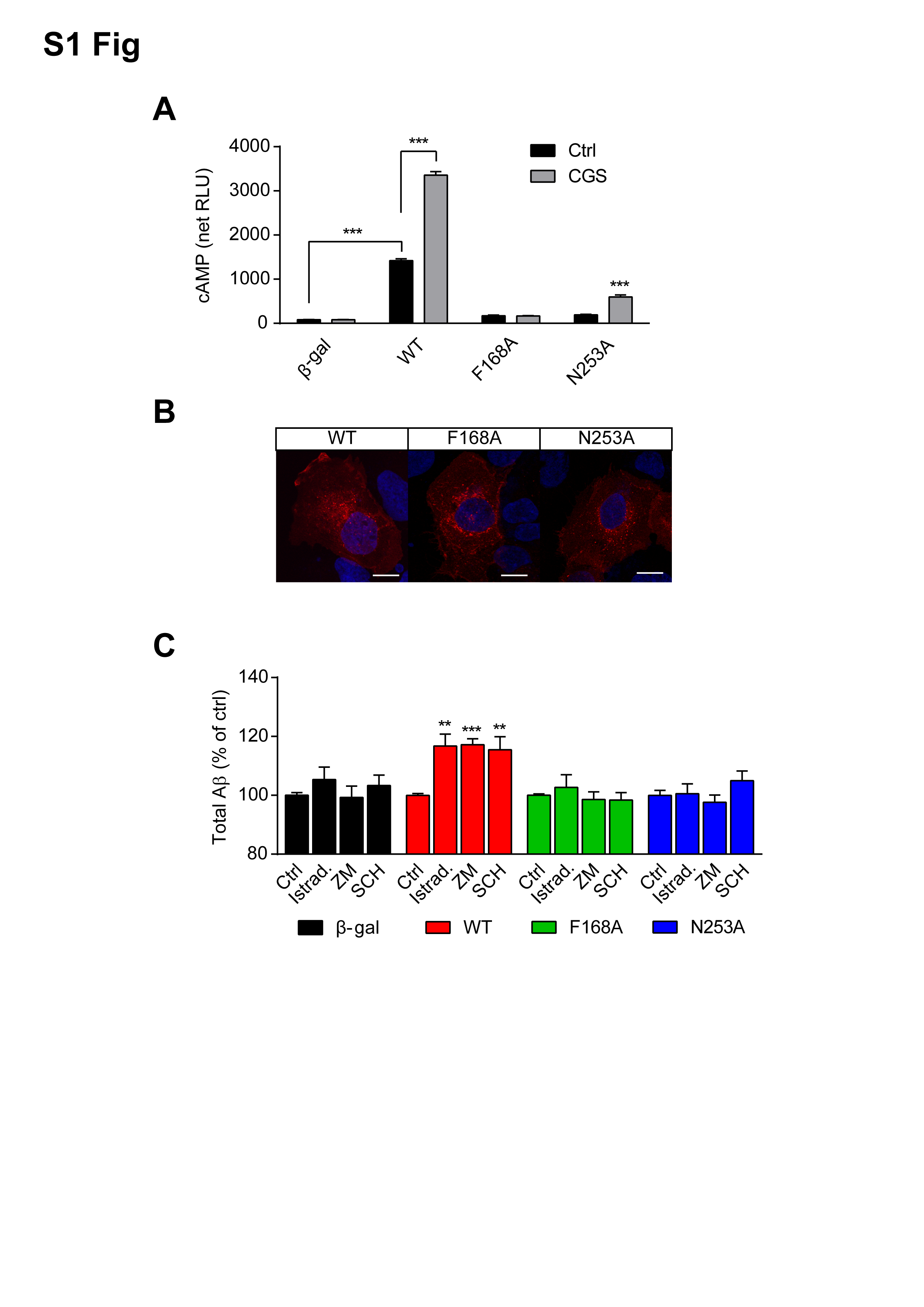

Supplement: S1 Fig — (A) The cAMP levels in CHO/APPswe cells over-expressing wild type or mutant A2ARs. Cells were co-transfected with pGloSensorTM-22F cAMP plasmid and β-galactosidase (β-gal), wild type-A2AR (WT), mutant F168A or N253A followed by the treatment with 0.1% of DMSO (Ctrl) or 30 nM of CGS 21680 HCl. (B) Representative image showing the cellular distribution of wild type-A2AR and its mutants in HEK293 cells. Cells were transfected with Flag-tagged wild type-A2AR (WT), F158A or N253A followed by immuno-staining. Scale bar = 10 μm. (C) ligand-modulated Aβ production in the cells expressing wild type-A2AR or its binding mutants. CHO/APPswe cells were transfected with β-galactosidase (β-gal), wild type-A2AR (WT), mutant F168A or N253A followed by the treatment with 0.1% of DMSO (Ctrl) or the indicated ligands at 30 nM. Data are representative or mean + SEM of at least three independent experiments. **, p < 0.01; ***, p < 0.001. (TIF) [file pone.0166415.s001.tif]

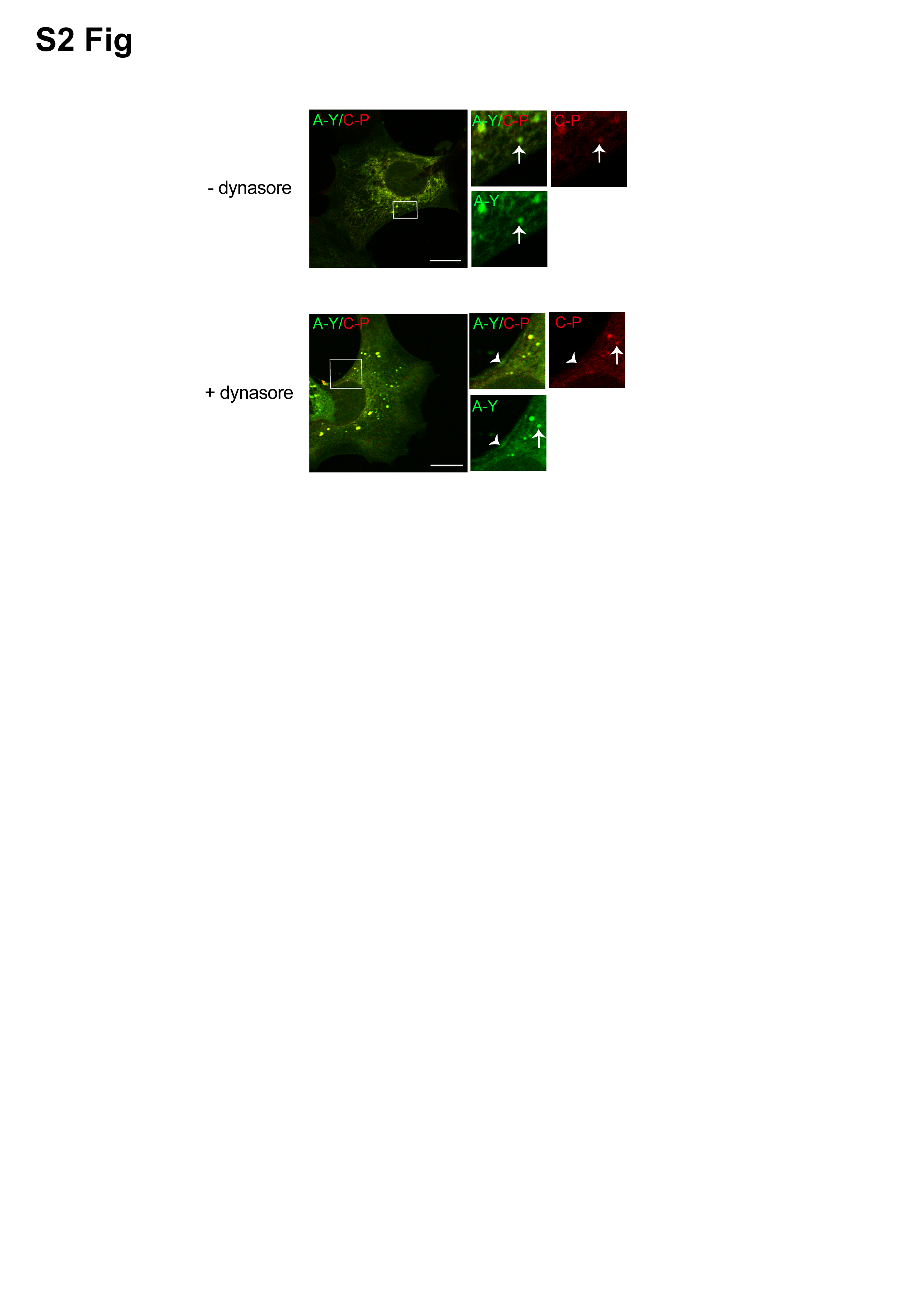

Supplement: S2 Fig — HEK293 cells were transfected with A2AR-YFP and γ-secretase components including CFP-PS1, NCT, APH1aL, and Pen2. Cells were then incubated without or with 80 μM dynasore for 30 min followed by fixation and imaging. The arrowheads and arrows indicate the expression at the plasma membrane and in the cytosol respectively. Scale bar = 10 μm. (TIF) [file pone.0166415.s002.tif]
